# Impaired lung function and mortality in Eastern Europe: results from multi-centre cohort study

**DOI:** 10.1186/s12931-022-02057-y

**Published:** 2022-05-31

**Authors:** Tatyana Court, Nadezda Capkova, Andrzej Pająk, Sofia Malyutina, Galina Simonova, Abdonas Tamosiunas, Martin Bobák, Hynek Pikhart

**Affiliations:** 1 Research Centre for Toxic Compounds in the Environment (RECETOX), Faculty of Science, Masaryk University, Koltarska 2, 611 37 Brno, Czech Republic; 2 Research Department of Epidemiology and Public Health, University College London, London, UK; 3 National Institute of Public Health, Prague, Czech Republic; 4 Department of Epidemiology and Population Sciences, Institute of Public Health, Jagiellonian University Medical College, Kraków, Poland; 5 Research Institute of Internal and Preventive Medicine – Institute of Cytology and Genetics, Siberian Branch of the Russian Academy of Sciences, Novosibirsk, Russia; 6 Novosibirsk State Medical University, Novosibirsk, Russia; 7 Laboratory of Population Research, Institute of Cardiology, Lithuanian University of Health Sciences, Kaunas, Lithuania

**Keywords:** Forced expiratory volume in one second, Pulmonary function test, Cohort study, Mortality

## Abstract

**Background:**

The association between impaired lung function and mortality has been well documented in the general population of Western European countries. We assessed the risk of death associated with reduced spirometry indices among people from four Central and Eastern European countries.

**Methods:**

This prospective population-based cohort includes men and women aged 45–69 years, residents in urban settlements in Czech Republic, Poland, Russia and Lithuania, randomly selected from population registers. The baseline survey in 2002–2005 included 36,106 persons of whom 24,993 met the inclusion criteria. Cox proportional hazards models were used to estimate the hazard ratios of mortality over 11–16 years of follow-up for mild, moderate, moderate-severe and very severe lung function impairment categories.

**Results:**

After adjusting for covariates, mild (hazard ratio (HR): 1.25; 95% CI 1.15‒1.37) to severe (HR: 3.35; 95% CI 2.62‒4.27) reduction in FEV1 was associated with an increased risk of death according to degree of lung impairment, compared to people with normal lung function. The association was only slightly attenuated but remained significant after exclusion of smokers and participants with previous history of respiratory diseases. The HRs varied between countries but not statistically significant; the highest excess risk among persons with more severe impairment was seen in Poland (HR: 4.28, 95% CI 2.14‒8.56) and Lithuania (HR: 4.07, 95% CI 2.21‒7.50).

**Conclusions:**

Reduced FEV1 is an independent predictor of all-cause mortality, with risk increasing with the degree of lung function impairment and some country-specific variation between the cohorts.

**Supplementary Information:**

The online version contains supplementary material available at 10.1186/s12931-022-02057-y.

## Background

Several previous studies have investigated the role of impaired lung function in risk of mortality [1–6]. It has been shown that reduced levels of forced expiratory volume measured in one second (FEV1) [3, 7, 8] and/or forced vital capacity (FVC) [9, 10] are good predictors of all-cause mortality in general population. Reduced pulmonary function was associated with mortality even among non-smokers without respiratory symptoms [9, 11] and at relatively modest levels of decrease in FEV1[12].

According to current the Global Initiative for Chronic Obstructive Lung Disease (GOLD) [13], grading the severity of lung function impairment from prediction equations (FEV1% predicted) derived from the population-based reference values is still recommended. Global Lung Function Initiative (GLI) equations [14] based on standardised Z-score has been also proposed, although, it has some limitations in elderly population [14, 15]. The assessment of lung function impairment by prediction equations is very challenging particularly in elderly population as it might lead to increased number of false positive results [16].

Most of the existing evidence in terms of mortality risk and lung function impairment is focused on Western Europe where exposure and risk factors may differ from the Central and Eastern European countries. In the urban rural epidemiology study (PURE), the decrease in FEV1 was associated with higher risk of mortality among people from low-income countries compared to population from middle or high incomes countries [12]. Since the 1970s, mortality rates have been considerably higher in Eastern Europe and the former Soviet Union [17, 18]: given this background of high mortality, the predictive power of lung function may differ from that reported in the Western Europe.

The prospective Health, Alcohol and Psychosocial factors in Eastern Europe (HAPIEE) cohort study has been designed to investigate risk factors for high rates of mortality and cardiovascular diseases in four Central and Eastern European countries (e.g., Czech Republic, Poland, Russia and Lithuania) [17]. It has been shown that low socioeconomic status was associated with impaired lung function in Poland [19]. To our knowledge, previous studies have not evaluated the risk of death associated with impaired lung function in these populations. In this study, we aimed to investigate the trend in FEV1 and FVC in association with all-cause mortality in the Eastern European populations in terms of level of lung function impairment and within countries variations.

## Methods

### Study design and participants

This study used data from the multinational prospective HAPIEE Project [17]. It includes randomly selected people with a mean age of 59 ± 7.3 years old from population registers in urban centres in Czech Republic, Poland, Russia and Lithuania (N = 36,106). The centres of data collection were located in seven towns in Czech Republic and in big cities such as Novosibirsk in Russia, Krakow in Poland and Kaunas in Lithuania. Baseline data on age, sex, health status, medical examination, lifestyle, socioeconomic and psychosocial factors were collected during 2002–2005. We used data from the baseline surveys conducted in 2002–2005 in the Czech Republic, Russia and Poland and in 2006–2008 in Lithuania. Data were collected using face-to-face interviews combined with the clinical examination, including spirometry.

The follow-up time was estimated based on deaths occurring until the end of 2020 in Czech Republic, until 31 July 2017 in Poland, until the end of 2017 in Russia and until 31 March 2019 in Lithuania. Persons with complete follow-up data were included in the study. Participants were censored on the date of death or the end of the study depending on data availability for each country.

All participants provided written informed consent. The study was performed in line with the principles of the Declaration of Helsinki. Approval was granted by the Joint UCL/UCLH Committees on the Ethics of Human Research (Committee Alpha), reference 99/0081; the Ethical Committee of the Institute of Internal Medicine, Siberian Branch of the Russian Academy of Medical Sciences, March 14, 2002 (Protocol No. 1); the Ethics Committee of the Kauans Medical University (reference P1-09/2005); and Ethics Committee at the National Institute of Public Health, Prague (reference 2002-01-08/P1).

### Spirometry and predicted values

Spirometry was performed using a Micro-Medical Microplus spirometer with the use of a standardised protocol and in accordance with the American Thoracic Society (ATS)/European Respiratory Society (ERS) recommended satisfactory repeatability criteria [20]. Participants with acute pulmonary infections and illnesses (e.g., vomiting and nausea), recent surgical procedures and cardiovascular conditions (e.g., myocardial infarction and stroke) were excluded from testing [20]. For each participant up to six pre-bronchodilator forced expiratory manoeuvres were performed. The attempts with the forced exhalation time for at least 6 s and without cough were accepted. The quality of the spirometry tests was ascertained by a qualified pulmonologist. Two or more measurements of forced expiratory volume in 1 s (FEV1) and forced vital capacity (FVC) within 150 ml variation considered for the study [20]. For each participant the highest values of FEV1 and FVC were selected for further analysis.

Predicted values of FEV1 and FVC were obtained for all participants with age and height as main predictors separately for men and women. Z-scores of FEV1 and FVC were calculated using the GLI equations [14]. A second set of predicted values was obtained from National Health and Nutrition Examination Survey (NHANES) III equations derived from Caucasian ethnic group of non-smoking healthy individuals considering the same age, sex and height groups [14, 21]. Both equations have shown a good ability to predict mortality [3, 16, 22]. Subsequently, predicted values of FEV1 and FVC were compared with observed values and the percentage of predicted value was calculated. The analysis was performed using %predicted cut-offs for the NHANES III equations and Z-score cut-offs from the GLI equations, these values were further categorised into normal (FEV1% > 80/Z-score > − 1.645), mild (FEV1% 80–70/− 1.645 > z-score > − 2), moderate (FEV1% 69–60/− 2 > Z-score > − 2.5), moderate-severe (FEV1% 59–50/− 2.5 > Z-score > − 3), severe (FEV1% 49–35/− 3 > Z-score > − 4), and very severe lung function impairment (FEV1% < 35/Z-score − 4) [23]. Similarly, the predicted values were obtained for FVC.

### Outcome

The primary outcome was all-cause mortality. Dates of death were obtained from the national or regional (Novosibirsk) death registers in each country. All registers have been shown a complete coverage of deaths [17].

### Covariates

Data on covariates was obtained from questionnaires and medical examination. The selection of variables was based on their known association with mortality [24, 25]. For the adjustment we considered age, sex, education (primary, secondary education, college or university degree), occupation (employed, retired or unemployed), deprivation scale (graded from 1 as a least deprived up to 12 as a most deprived), smoking status (never, current or past heavy smoker (> 30 cigarettes per day), moderate smoker (11–29 cigarettes per day), or light smoker (< 10 cigarettes per day)) [26], alcohol consumption (never, graduated frequency from 1 to 3 drinks monthly or 1–5 drinks weekly), physical activity ( as number of hours demanding physical activity per week) and healthy diet score [27]. We also identified the following self-reported comorbidities: stroke, myocardial infarction, ischemic heart disease, hypertension (defined as measured blood pressure > 140/90 mm Hg and/or self-reported treated hypertension), diabetes (treated and/or untreated), asthma and chronic obstructive pulmonary disease (COPD), any type of surgery (in last 3 months) and cancer.

Information on pulmonary symptoms such as cough with or without phlegm (for 3 months) and chest pain were also included in the analyses as markers of respiratory diseases.

Confounders obtained during medical examination were weight, height, body-mass index (BMI), blood pressure.

### Statistical analyses

All analyses were performed with Stata (Version 14; StataCorp). Descriptive statistics are presented as means with standard deviations (SD) or frequencies with proportions.

The association of FEV1 and FVC categories with the risk of death was estimated using Cox proportional hazards regression models. FEV1 and FVC predicted values were entered into the model as categorical variables accounting for level of lung function impairment with the “no impairment” category as a reference. We used robust variance estimator to account for possible interactions between groups and multiple comparison. Proportional hazards assumptions were confirmed by exploring parallelism of log negative and log estimated survival curves for each covariate (Additional file 1: Fig. S1). Hazard ratios (HR) with their corresponding 95% confidence intervals (CI) were estimated by crude (included age and sex) and confounder-adjusted models.

The main analyses included smokers and non-smokers as well as people with previous history of respiratory diseases. In order to exclude potential influence of these confounders on mortality risk, we conducted sensitivity analyses after excluding people with smoking history and history of chronic respiratory diseases (e.g., COPD and asthma) and symptoms.

We also conducted separate stratified sensitivity analysis for each country with different follow-up time.

## Results

**Fig. 1:**
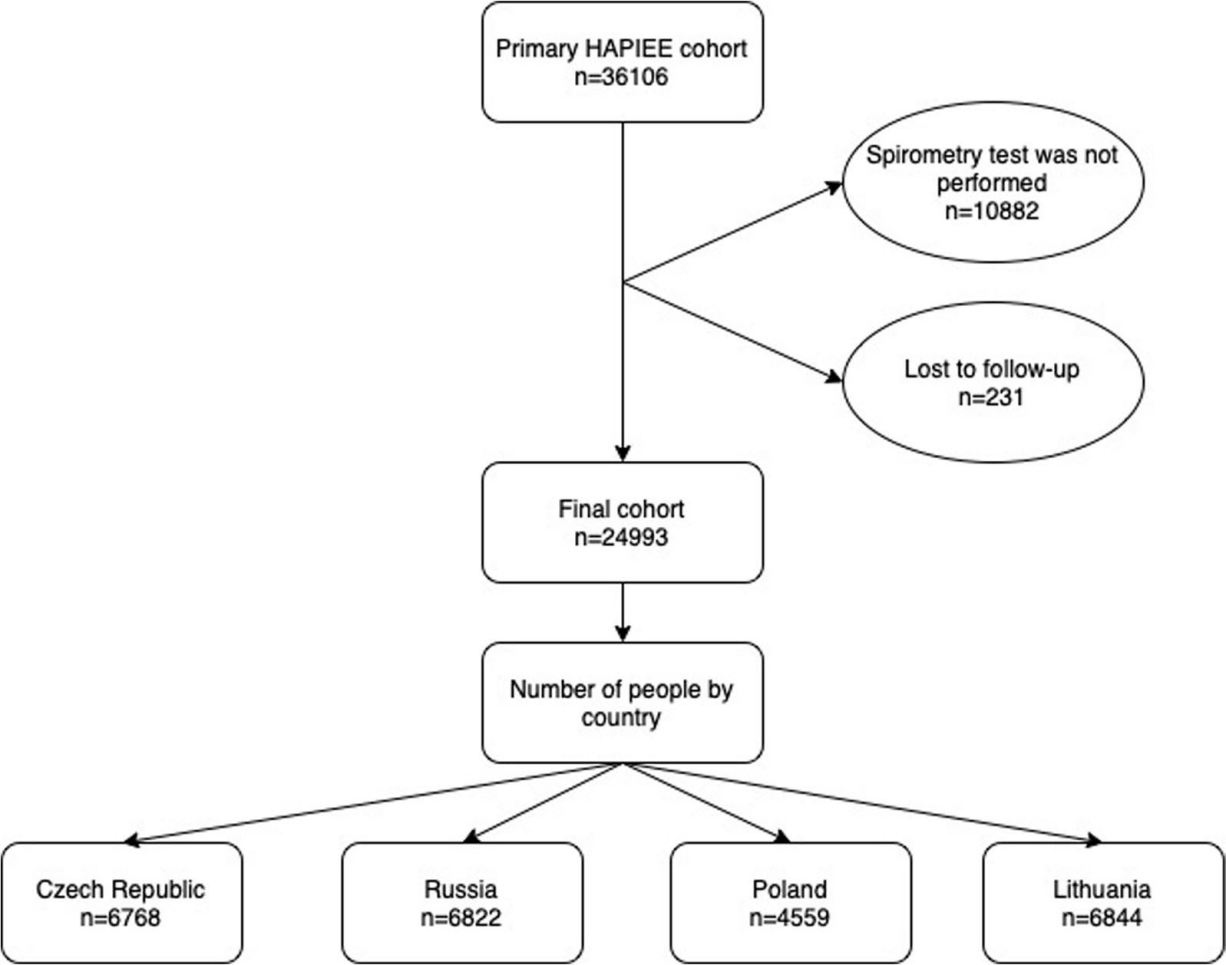
Flowchart of exclusion criteria of the HAPIEE study cohort

Altogether 36,106 individuals were recruited at baseline, of whom 24,993 met the inclusion criteria (Fig. 1). Spirometry was conducted on 25,224 persons. The relatively large number of persons with missing spirometry data was due to non-response in clinical examination in Czech and Polish participants. In addition, spirometry tests in the Polish cohort were only done on random 50% of respondents in the 2^nd^ year of baseline survey for logistic reasons. Compared to people with normal spirometry values, people with abnormal FEV1 were older with increasing proportion of men, people with smoking history and with chronic cardiovascular and lung diseases (Additional file 1: Table S1, S2).

In comparison between countries, Russian participants were more likely to have a history of smoking and the proportion of people with severe lung impairment was the highest (Table 1). In contrast, participants from Poland sample were more likely to have a history of cardiovascular and metabolic diseases including ischemic heart disease, myocardial infarction and diabetes. COPD with respiratory symptoms was more common in population from Lithuania; and higher frequency of alcohol use was observed in people from Czech Republic (Table 1).

**Table 1:** Characteristics of the study sample by country (n = 24 993)

	Czech Republic	Russia	Poland	Lithuania
	(n = 6 768)	(n = 6 882)	(n = 4 559)	(n = 6 844)
Age (years), mean (SD)	58.3 (7.1)	57.9 (7.0)	58.1 (6.9)	60.9 (7.6)
Age (years), %				
< 50	16.6	17.3	16.2	11.9
50–59	37.7	43.2	42.5	31.8
60–69	44.0	38.0	39.4	43.3
≥ 70	1.7	1.5	1.9	13.0
Women, %	54.2	54.0	50.6	54.5
Occupational status, %				
Employed	43.4	36.6	35.8	39.7
Retired/employed	8.0	18.5	5.9	17.5
Retired/unemployed	45.2	39.4	51.9	37.2
Unemployed	3.4	5.5	6.4	5.6
Smoking status, %				
Current, ≥ 1 cigarette	23.0	28.2	28.3	17.4
Current, < 1 cigarette	2.7	1.0	2.0	2.1
Past smoker	29.7	13.4	28.2	17.9
Never	44.6	57.3	41.5	62.6
Smoking category^a^, %				
Light	55.4	36.3	43.7	45.6
Moderate	38.2	51.2	44.1	45.2
Heavy	6.4	12.5	12.2	9.2
Alcohol consumption^b^, %				
Never	11.7	14.5	35.1	47.4
< 1/monthly	25.9	37.6	21.8	26.7
1–3/monthly	21.1	21.6	0.2	19.9
1–4/weekly	28.5	23.5	19.1	5.5
≥ 5/weekly	12.9	2.7	3.8	0.5
Deprivation range^c^, mean (SD)	1.6 (2.3)	3.8 (3.6)	2.1 (2.9)	1.0 (1.9)
Physical activity moderate^d^, mean (SD)	13.7 (12.2)	16.8 (11.7)	13.9 (10.6)	15.8 (10.8)
Physical activity vigorous^e^, mean (SD)	4.4 (5.4)	2.5 (5.9)	5.6 (6.1)	2.9 (4.8)
BMI, mean (SD), kg/m^2^	28.2 (4.6)	28.5 (5.5)	28.3 (4.6)	29.4 (5.3)
*Comorbidities, %*				
Cardiovascular diseases				
Hypertension	65.2	65.6	60.5	66.5
Myocardial infarction	5.0	7.2	8.3	7.8
Ischemic heart disease	8.2	15.7	19.0	9.8
Stroke	3.3	4.8	2.5	4.2
Lung diseases				
COPD	14.4	23.3	11.3	16.3
Asthma	4.6	3.0	6.8	3.9
Cough (> 3 months)	14.0	17.5	16.6	14.8
Chest pain (> 3 months)	12.5	15.5	15.3	15.5
Any type of cancer	6.3	2.9	5.1	7.2
Other diseases				
Diabetes	11.4	5.2	12.1	7.6
Any type of surgery	2.0	0.2	1.9	1.0
Spirometry, mean (SD)				
FEV1	2.55 (0.8)	2.58 (0.8)	2.55 (0.8)	2.63 (0.8)
FVC	3.3 (0.9)	3.1 (0.9)	3.1 (0.9)	3.3 (0.9)
FEV1predicted (NHANES)^f^	3.0 (0.6)	2.86 (0.6)	2.92 (0.6)	2.85 (0.6)
Z-score^g^	− 0.56 (1.1)	− 0.34 (1.2)	− 0.54 (1.1)	− 0.14 (1.1)

BMI, body mass index; COPD, chronic obstructive pulmonary disease; FEV1, forced expiratory volume in 1 s; FVC, forced vital capacity

^a^Smoking category ((current or past heavy (> 30 cigarettes per day), moderate (11–29 cigarettes per day), or light (< 10 cigarettes per day))

^b^Alcohol consumption (never, graduated frequency from 1 to 3 drinks monthly or 1–5 drinks weekly)

^c^Deprivation scale (graded from 1 as a least deprived up to 12 as a most deprived)

^d^Number of hours per week undertaken by household domain physical activity (e.g., housework, gardening, maintenance of the house etc.)

^e^Number of hours of vigorous physical activity per week (e.g., sports, play games and hiking)

^f^National Health and Nutrition Examination Survey (NHANES) III equations

^g^The reference values from the Global Lung Initiative (GLI) with threshold point below lower limit of normal (-1.645)

The proportion of people with abnormal FEV1 based on NHANES III algorithm were higher compared with those obtained from GLI 2012 Eqs. (4453 vs 3095) although the risk of death showed a similar trend in comparison groups categorised by both methods (Table 2).

**Table 2:** Association between degree of lung function impairment and all-cause mortality by type of FEV1 predicted

Type of groups	No. of persons	No. of deaths	Person-years of follow-up	Deaths per 100 person-years (95% CI)	Model 1 adjusted HR^†^ (95% CI)	Model 2 Adjusted HR^‡^ (95% CI)
FEV1% predicted impairment groups (NHANES)*						
Normal	20,506	3632	267,620	1.36 (1.31–1.40)	1.00	1.00
Mild	2452	700	31,350	2.23 (2.07–2.40)	1.55 (1.43–1.68)	1.25 (1.15–1.37)
Moderate	1068	394	12,879	3.06 (2.77–3.38)	2.01 (1.80–2.24)	1.51 (1.35–1.69)
Moderate-severe	487	219	5465	4.01 (3.51–4.57)	2.51 (2.17–2.91)	1.82 (1.58–2.11)
Severe	342	163	3814	4.27 (3.67–4.98)	2.55 (2.16–3.02)	1.82 (1.54–2.15)
Very severe	104	69	961	7.18 (5.67–9.09)	4.44 (3.36–5.87)	3.35 (2.62–4.27)
FEV1 impairment groups (Z-score)^§^						
Normal	21,864	4045	284,792	1.42 (1.38–1.46)	1.00	1.00
Mild	1052	315	13,302	2.37 (2.12–2.64)	1.65 (1.47–1.85)	1.27 (1.13–1.43)
Moderate	923	310	11,284	2.75 (2.45–3.07)	1.83 (1.63–2.06)	1.41 (1.25–1.59)
Moderate-severe	523	226	6002	3.77 (3.30–4.29)	2.48 (2.16–2.86)	1.81 (1.57–2.09)
Severe	471	214	5367	3.99 (3.49–4.56)	2.55 (2.20–2.95)	1.87 (1.62–2.16)
Very severe	126	67	1342	4.99 (3.93–6.35)	4.50 (3.45–5.86)	3.42 (2.67–4.37)

FEV1, forced expiratory volume in 1 s; CI, confidence interval; HR, hazard ratio

^*^Sex-specific predicted values of FEV1 (FEV1%) standardized for age and height based on NHANES III equations

^*§*^The reference values from the GLI 2012 z-score with threshold point below lower limit of normal (− 1.645)

^†^Adjusted for age, sex and country

^‡^Adjusted for age, sex, occupation and education; alcohol consumption, smoking status, level of physical activity and body mass index; history of hypertension, ischemic heart disease, myocardial infarction, stroke

In total, 5211 persons died during the average of 13 years of follow-up (Additional file 1: Table S3). Mortality rate showed gradual increase associated with the degree of lung function impairment and the rate ranged from 2.23 (95% confidence interval (CI) 2.07‒2.40) per 100 person-years among people with mild impairment to 7.18 (95% CI 5.67‒9.09) per 100 person-years among people with very severe lung function impairment compared to healthy individuals (mortality rate 1.36, 95% CI 1.31–1.40 per 100 person-years) (Table 2). In the Cox proportional hazards regression model adjusted for age and sex, low FEV1 was associated with a 55% increased relative risk of death (HR 1.55, 95% CI 1.43‒1.68) in people with mild and more than four-fold increase (HR 4.44, 95% CI 3.36‒5.87) in people with very severe lung function impairment compared with participants with no impairment. When the model was adjusted for other baseline covariates, the HR decreased to 1.25 (95% CI 1.15‒1.37) in the group with mild and to 3.35 (95% CI 2.62‒4.27) in very severe lung function impairment.  Similar results were observed with FVC% indices, therefore, further analyses were conducted with FEV1% (Additional file 1: Table S4).

The association between decrease in FEV1 even to mild degree and all-cause mortality was still observed after exclusion of people with previous history of respiratory diseases and symptoms (Table 3, HR 1.20, 95% CI 1.07‒1.35) as well as among never smokers (HR 1.40, 95% CI 1.21‒1.62) (Table 3) (Additional file 1: Table S4).

**Table 3:** Association between degree of lung function impairment and all-cause mortality restricted to people without previous history of COPD and never smokers

Type of groups	No. of persons	No. of deaths	Person-years of follow-up	Deaths per 100 person-years (95% CI)	Model 1 adjusted HR^†^ (95% CI)	Model 2 Adjusted HR^‡^ (95% CI)
*No previous history of COPD and pulmonary symptoms*						
FEV1% predicted impairment groups*						
Normal	14,436	2413	190,254	1.27 (1.22–1.32)	1.00	1.00
Mild	1427	374	18,736	2.00 (1.80–2.21)	1.51 (1.35–1.68)	1.20 (1.07–1.35)
Moderate	516	175	6463	2.71 (2.33–3.14)	1.88 (1.60–2.21)	1.41 (1.20–1.66)
Moderate-severe	196	76	2337	3.25 (2.60–4.07)	2.12 (1.64–2.73)	1.59 (1.26–2.02)
Severe	117	38	1463	2.60 (1.89–3.57)	1.83 (1.30–2.57)	1.30 (0.93–1.82)
Very severe	31	12	360	3.34 (1.89–5.87)	3.07 (1.68–5.61)	2.13 (1.21–3.77)
*Never smokers*						
FEV1% predicted impairment groups*						
Normal	11,233	1537	147,841	1.04 (0.99–1.09)	1.00	1.00
Mild	1077	246	14,057	1.75 (1.54–1.98)	1.56 (1.37–1.79)	1.40 (1.21–1.62)
Moderate	407	111	5179	2.14 (1.78–2.58)	1.89 (1.55–2.30)	1.61 (1.32–1.98)
Moderate-severe	176	59	2096	2.81 (2.18–3.63)	2.50 (1.89–3.31)	2.17 (1.66–2.83)
Severe	104	33	1294	2.55 (1.81–3.59)	2.39 (1.65–3.45)	2.14 (1.50–3.07)
Very severe	36	19	397	4.79 (3.06–7.51)	4.06 (2.43–6.80)	4.03 (2.55–6.35)

FEV1, forced expiratory volume in 1 s; CI, confidence interval; HR, hazard ratio

^*^Sex-specific predicted values of FEV1 (FEV1%) standardized for age and height based on NHANES III equations

^†^Adjusted for age, sex and country

^‡^Adjusted for age, sex, occupation and education; alcohol consumption, smoking status, level of physical activity and body mass index; history of hypertension, ischemic heart disease, myocardial infarction, stroke

When the analyses were stratified by country, the association between very severe lung function impairment and risk of death was lower in Czech Republic (HR 2.99, 95% CI 1.76‒5.09) and Russia (HR 2.98, 95% CI 2.07‒4.30), while in Poland (HR 4.28, 95% CI 2.14‒8.56) and Lithuania (HR 4.07, 95% CI 2.21‒7.50) the excess risk of death in the very severe impaired group was highest (Table 4). Although, the confidence intervals were wider and the differences in HRs between countries were not statistically significant (p = 0.86). Similar results were obtained with balanced follow-up time (restricted to 10 years) (Additional file 1: Table S5).

**Table 4:** Association between degree of lung function impairment (FEV1% predicted) and all-cause mortality by country

Type of groups	No. of persons	No. of deaths	Person-years of follow-up	Deaths per 100 person-years (95% CI)	Model 1 adjusted HR^†^ (95% CI)	Model 2 adjusted HR^‡^ (95% CI)
*Czech Republic*						
FEV1% predicted impairment groups*						
Normal	5283	1081	84,319	1.28 (1.21–1.36)	1.00	1.00
Mild	831	268	12,636	2.12 (1.88–2.39)	1.54 (1.34–1.76)	1.31 (1.13–1.52)
Moderate	366	140	5347	2.62 (2.22–3.09)	1.71 (1.43–2.05)	1.25 (1.02–1.53)
Moderate-severe	135	56	1915	2.92 (2.25–3.80)	2.01 (1.52–2.67)	1.49 (1.10–2.01)
Severe	110	49	1469	3.33 (2.52–4.41)	2.36 (1.73–3.21)	1.85 (1.34–2.53)
Very severe	29	18	357	5.04 (3.17–8.00)	3.53 (2.18–5.72)	2.99 (1.76–5.09)
*Russia*						
FEV1% predicted impairment groups*						
Normal	5621	1114	70,294	1.58 (1.49–1.68)	1.00	1.00
Mild	595	180	7060	2.55 (2.20–2.95)	1.39 (1.19–1.63)	1.11 (0.95–1.31)
Moderate	262	106	2810	3.77 (3.12–4.56)	2.15 (1.75–2.64)	1.68 (1.37–2.06)
Moderate-severe	163	75	1699	4.42 (3.52–5.54)	2.31 (1.79–2.99)	1.69 (1.33–2.15)
Severe	116	56	1239	4.52 (3.48–5.87)	2.11 (1.59–2.80)	1.61 (1.22–2.11)
Very severe	46	31	390	7.96 (5.60–11.31)	3.63 (2.36–5.58)	2.98 (2.07–4.30)
*Poland*						
FEV1% predicted impairment groups*						
Normal	3650	533	46,585	1.14 (1.05–1.25)	1.00	1.00
Mild	504	111	6188	1.79 (1.49–2.16)	1.53 (1.25–1.87)	1.20 (0.96–1.50)
Moderate	234	66	2786	2.37 (1.86–3.01)	1.98 (1.53–2.56)	1.63 (1.24–2.13)
Moderate-severe	97	45	1026	4.39 (3.27–5.87)	3.38 (2.46–4.64)	2.40 (1.73–3.33)
Severe	59	29	597	4.85 (3.37–6.99)	3.08 (2.06–4.61)	2.12 (1.39–3.24)
Very severe	14	9	120	7.48 (3.89–14.37)	6.95 (3.49–13.84)	4.28 (2.14–8.56)
*Lithuania*						
FEV1% predicted impairment groups*						
Normal	5952	904	66,422	1.36 (1.28–1.45)	1.00	1.00
Mild	522	141	5467	2.58 (2.19–3.04)	1.87 (1.56–2.24)	1.50 (1.25–1.81)
Moderate	206	82	1935	4.24 (3.41–5.26)	2.84 (2.24–3.60)	2.04 (1.57–2.65)
Moderate-severe	92	43	825	5.21 (3.86–7.02)	2.83 (2.00–3.99)	2.18 (1.59–2.99)
Severe	57	29	508	5.71 (3.96–8.21)	3.00 (2.02–4.46)	2.01 (1.37–2.94)
Very severe	15	11	94	11.71 (6.48–21.14)	5.79 (2.54–13.19)	4.07 (2.21–7.50)

FEV1, forced expiratory volume in 1 s; CI: confidence interval; HR: hazard ratio

^*^Sex-specific predicted values of FEV1 (FEV1%) standardized for age and height based on NHANES III equations

^†^Adjusted for age and sex

^‡^Adjusted for age, sex, occupation and education; alcohol consumption, smoking status, level of physical activity and body mass index; history of hypertension, ischemic heart disease, myocardial infarction, stroke

## Discussion

In this study of 24,993 persons from four Central and Eastern European countries, we found an independent, strong and dose-dependent association between lung function impairment and all-cause mortality. The same relationship was observed even if the sample was restricted to never-smokers and after exclusion of persons with history of respiratory diseases. The comparison between countries suggested a some non-statistically significant variation in the strength of the association with strongest associations seen in Poland and Lithuania. Similar trend was also observed in country-specific comparisons with balanced follow-up time.

The HAPIEE study was designed to investigate possible risk factors linked to mortality in Eastern Europe and former Soviet Union, a region with high and rising mortality due to economic and political crisis [18]. Given this health pattern, identification of potential predictors of mortality in this region is pivotal. Association of reduced spirometry indices with overall mortality has been well established in western populations [1, 5, 6, 8, 28]. The findings that all-cause mortality increased with the severity of lung function impairment are in accordance with a previous cohort study of elderly individuals [3]. In our study, the excess risk was large, with more than doubled mortality in the group of severe compared to mild lung function impairment, although our results for the group with severe lung function impairment are based on relatively small number of study participants. This might be explained by differences in grading criteria of commonly used FEV1 thresholds of lung function in three stages of impairment rather than 5 stages criteria; and by the difference in methods where they assessed the difference between stages in fixed time-points rather than as a continuous exposure.

In our study, the excess risk of death associated with impaired lung function remained high even after exclusion of people with previous history of smoking and respiratory diseases. Similar pattern has been reported in the survey-based cohort study of lifelong non-smokers [9]. In that study, the decline in FEV1 was associated with mortality with stronger effect than common risk factors (e.g., increased blood pressure or obesity). It might be possible that impaired lung function shares pathophysiological mechanisms and risk factors with other chronic conditions with established mortality burden such as cardiovascular disease and diabetes [29, 30]. The fact that low FEV1 predicted mortality even in relatively healthy individuals with mild impairment and without smoking history could indicate the importance of lung function in prediction (and prevention) of common chronic diseases and mortality risk.

As the majority of studies so far have been performed in high income countries of Western Europe and North America, there is a paucity of studies on the risk of death associated with impaired lung function in other populations. In the large prospective international cohort study among people from urban and rural communities, the mortality risk was increased in all lung function impairment groups but it remained higher among people from low and middle income countries in comparison to high-income populations [12]. In that study, the mortality rates in low-income countries from that study were similar to our pooled results. In our study, the inverse graded association between reduced FEV1 and mortality was observed in all four countries. The results were similar across countries, there was some (not statistically significant) variation in the strength of the association, with higher hazard ratios in Poland and Lithuania for severe lung function impairment. Similar results were observed after balancing follow-up time. These results might be explained by differences in socioeconomic status and health behaviour patterns between countries. In the previous HAPIEE study [18], larger socioeconomic inequalities in association with mortality among four countries were observed in the Russian population; however, we did not find stronger effect of lung function impairment in Russia. Unmeasured differences in health status are the most likely explanation of our results; e.g., subjects from Poland and Lithuania had higher proportion of cardiovascular, chronic lung conditions and diabetes.

### Strengths and limitations

Our study included people from urban communities of four Central and Eastern European countries. While the study cohort is in general representative for urban populations, it does not include rural areas, and the results are thus not generalizable to entire population of included countries. The response rate was comparable and follow-up time was balanced between countries. The large number of investigating covariates to be adjusted in the analyses of lung function impairment and mortality is the particular strength of this study.

Assessment of predictive values using two system approach is a strength and supports our results. In our study we did not observe major differences in mortality trend between grading of lung function based on NHANES III algorithm and (GLI) 2012 z-score equations. The number of people with impaired lung function identified with NHANES III algorithm was higher and it might be that some results were false positive. Data on normal spirometry indices from old persons is lacking and both methods might lead to misclassification of lung function in this age group [14]. However, compared to GLI 2012 z-score, NHANES III equations are considered as a better predicting tool for elderly population [14–16, 31].

Self-reported information in the questionnaire is subjective to recall bias. The fact that almost 20% of Czech and Polish participants were less healthy and did not underwent the baseline clinical examination might lead to the underestimation of our results.

The numbers of people in severe impairment groups were too small for investigations with sufficient statistical power, therefore, the comparison results in these groups should be interpreted with caution.

Finally, the nature of our design cannot entirely prove causality, although the longitudinal design, extensive covariate adjustment of models and long follow-up likely to minimize these limitations.

## Conclusions

This is first study investigating the role of impaired lung function in association with mortality in the population of four Central and Eastern European countries. The results are similar across countries with distinct socioeconomic and mortality risk profile and in line with previously reported findings from western countries. Low FEV1 consistently predicted mortality with a clear dose–response fashion. The associations remained strong among non-smokers and in individuals with no history of respiratory disease. These findings advocate for implementing spirometry into the risk prediction systems. This would improve the identification of people at increased risk of death, and more intensive efforts in primary or secondary prevention can be introduced in this group.

## Supplementary Information

**Additional file 1: Table S1.** Characteristics of the study sample by degree of lung function impairment based on (NHANES) III equations (n=24 993). **Table S2.** Characteristics of the study sample by degree of lung function impairment based on Z-score (n=24 993). **Table S3.** Survival data by country. **Table S4.** Association between degree of lung function impairment and all-cause mortality by type of FVC predicted. **Table S5.** Association between degree of lung function impairment (FEV1% predicted) and all-cause mortality by country (follow-up restricted to 10 years). **Figure S1.** Kaplan–Meier survival curves by groups of lung function impairment.

## Data Availability

The data used to conduct the research are available from the corresponding author but restrictions by the register maintainers apply to the availability of these data. Therefore, the data are not publicly available. However, data are available from the authors upon reasonable request and with permission of the register maintainers.

## Abbreviations

HAPIEE: Health, Alcohol and Psychosocial factors in Eastern Europe
FEV1: Forced expiratory volume measured in one second
FVC: Forced vital capacity
FEV1% predicted: Percent predicted values of forced expiratory volume in one second
FVC% predicted: Percent predicted values of forced vital capacity
GOLD: Global Initiative for Chronic Obstructive Lung Disease
GLI: Global Lung Function Initiative
NHANES III: National Health and Nutrition Examination Survey
COPD: Chronic obstructive pulmonary disease
HR: Hazard ratio
SD: Standard deviation
CI: Confidence interval
