# Supplementary material for: Impaired lung function and mortality in Eastern Europe: results from multi-centre cohort study
Source: Respir Res. 2022 May 31;23:140. doi: 10.1186/s12931-022-02057-y (PMC9153198; doi:10.1186/s12931-022-02057-y)

**Table S1**. **Characteristics of the study sample by degree of lung function impairment based on (NHANES) III equations (n=24 993)**

|  | **Normal** | **Mild** | **Moderate** | **Moderate-severe** | **Severe** | **Very severe** |
| --- | --- | --- | --- | --- | --- | --- |
|  | **(n=20 536)** | **(n=2 452)** | **(n=1 072)** | **(n=487)** | **(n=342)** | **(n=104)** |
|  |  |  |  |  |  |  |
| Age (years), mean (SD) | 58.5 (7.3) | 59.8 (6.9) | 60.5 (6.9) | 61.2 (6.9) | 61.6 (6.8) | 61.5 (6.9) |
| Age (years), % |  |  |  |  |  |  |
| *< 50* | 16.7 | 10.7 | 9.2 | 8.2 | 8.2 | 6.7 |
| *50-59* | 39.1 | 37.7 | 34.1 | 32.0 | 29.8 | 29.8 |
| *60-69* | 39.5 | 47.4 | 50.6 | 52.4 | 56.1 | 57.7 |
| *≥ 70* | 4.7 | 4.2 | 6.2 | 7.4 | 5.9 | 5.8 |
| Women, % | 53.9 | 55.1 | 51.1 | 48.9 | 42.7 | 39.4 |
| Country, % |  |  |  |  |  |  |
| *Czech Republic* | 25.8 | 33.9 | 34.4 | 27.7 | 32.2 | 27.9 |
| *Russia* | 27.4 | 24.3 | 24.5 | 33.5 | 33.9 | 44.2 |
| *Poland* | 17.8 | 20.6 | 21.8 | 19.9 | 17.2 | 13.5 |
| *Lithuania* | 29.0 | 21.3 | 19.2 | 18.9 | 16.7 | 14.4 |
| Occupational status, % |  |  |  |  |  |  |
| *Employed* | 41.6 | 31.1 | 27.4 | 18.1 | 20.9 | 17.3 |
| *Retired/employed* | 13.6 | 11.6 | 10.5 | 13.2 | 8.5 | 4.8 |
| *Retired/unemployed* | 39.8 | 52.4 | 56.3 | 62.1 | 65.3 | 72.1 |
| *Unemployed* | 5.0 | 4.9 | 5.8 | 6.6 | 5.3 | 5.8 |
| Smoking status, % |  |  |  |  |  |  |
| *Current, ≥ 1 cigarette* | 21.6 | 31.8 | 36.9 | 34.6 | 43.5 | 44.2 |
| *Current, < 1 cigarette* | 2.0 | 1.6 | 1.9 | 3.3 | 0.6 | 2.9 |
| *Past smoker* | 21.4 | 22.4 | 22.8 | 25.9 | 25.4 | 18.3 |
| *Never* | 55.0 | 44.2 | 38.3 | 36.2 | 30.4 | 34.6 |
| Smoking category^a^, % |  |  |  |  |  |  |
| *Light* | 48.0 | 41.3 | 40.5 | 36.1 | 36.6 | 28.8 |
| *Moderate* | 42.8 | 46.4 | 47.7 | 50.6 | 51.8 | 63.6 |
| *Heavy* | 9.2 | 12.3 | 11.8 | 13.3 | 11.5 | 7.6 |
| Alcohol consumption^b^, % |  |  |  |  |  |  |
| *Never* | 26.0 | 30.3 | 28.1 | 30.3 | 27.4 | 31.7 |
| *<1/monthly* | 28.9 | 26.6 | 27.3 | 28.7 | 28.0 | 25.0 |
| *1-3/monthly* | 21.1 | 19.2 | 18.7 | 17.5 | 18.6 | 17.3 |
| *1-4/weekly* | 19.2 | 18.4 | 18.9 | 17.7 | 20.3 | 21.1 |
| *≥5/weekly* | 4.8 | 5.5 | 6.9 | 5.8 | 5.6 | 4.8 |
| Deprivation range^c^, mean (SD) | 2.1 (2.9) | 2.4 (3.1) | 2.6 (3.3) | 2.9 (3.5) | 2.9 (3.3) | 3.4 (3.1) |
| Physical activity moderate^d^, mean (SD) | 15.4 (11.5) | 14.7 (11.3) | 14.1 (12.0) | 13.7 (10.9) | 14.2 (12.5) | 14.7 (12.4) |
| Physical activity vigorous^e^, mean (SD) | 3.7 (5.6) | 3.6 (5.7) | 3.3 (5.3) | 3.5 (6.1) | 3.6 (6.8) | 2.6 (5.1) |
| BMI, mean (SD), kg/m^2^ | 28.5 (4.8) | 29.6 (5.9) | 29.4 (5.8) | 29.6 (6.4) | 28.3 (5.9) | 27.0 (6.5) |
| **Comorbidities, %** |  |  |  |  |  |  |
| ***Cardiovascular diseases*** |  |  |  |  |  |  |
| *Hypertension* | 63.2 | 70.8 | 73.5 | 75.9 | 75.5 | 66.3 |
| *Myocardial infarction* | 6.3 | 8.9 | 12.4 | 12.1 | 10.6 | 15.4 |
| *Ischemic heart disease* | 11.8 | 15.0 | 18.7 | 21.2 | 16.3 | 23.3 |
| *Stroke* | 3.4 | 5.5 | 6.6 | 4.6 | 5.6 | 6.9 |
| ***Lung diseases*** |  |  |  |  |  |  |
| *COPD* | 14.2 | 21.9 | 32.5 | 39.2 | 46.6 | 49.5 |
| *Asthma* | 2.9 | 7.3 | 12.5 | 20.2 | 21.8 | 21.4 |
| *Cough (>3 months)* | 13.2 | 21.0 | 27.6 | 35.8 | 47.4 | 44.2 |
| *Chest pain (>3 months)* | 12.7 | 18.4 | 25.4 | 29.8 | 42.5 | 36.5 |
| ***Any type of cancer*** | 5.3 | 5.0 | 7.4 | 6.7 | 6.8 | 3.9 |
| ***Other diseases*** |  |  |  |  |  |  |
| *Diabetes* | 7.8 | 13.1 | 14.3 | 15.0 | 12.6 | 3.9 |
| *Any type of surgery* | 1.1 | 1.3 | 1.6 | 1.1 | 0.6 | 2.9 |
| ***Spirometry, mean (SD)*** |  |  |  |  |  |  |
| *FEV1* | 2.8 (0.7) | 2.0 (0.5) | 1.7 (0.4) | 1.4 (0.4) | 1.1 (0.3) | 0.7 (0.2) |
| *FVC* | 3.3 (0.8) | 2.7 (0.7) | 2.5 (0.8) | 2.3 (0.8) | 2.2 (0.8) | 1.9 (1.0) |
| *FEV1predicted (NHANES)^f^* | 2.9 (0.6) | 2.9 (0.6) | 2.9 (0.6) | 2.8 (0.6) | 2.8 (0.6) | 2.8 (0.6) |

BMI, body mass index; COPD, chronic obstructive pulmonary disease; FEV1, forced expiratory volume in 1 second; FVC, forced vital capacity.

^a^ Smoking category ((current or past heavy smoker (>30 cigarettes per day), moderate smoker (11 - 29 cigarettes per day), or light smoker (<10 cigarettes per day)).

^b^ Alcohol consumption (never, graduated frequency from 1-3 drinks monthly or 1-5 drinks weekly).

^c^Deprivation scale (graded from 1 as a least deprived up to 12 as a most deprived).

^d^Number of hours per week undertaken by household domain physical activity (e.g., housework, gardening, maintenance of the house etc).

^e^Number of hours of vigorous physical activity per week (e.g., sports, play games and hiking)

^f^Extrapolating National Health and Nutrition Examination Survey (NHANES) III equations.

**Table S2**. **Characteristics of the study sample by degree of lung function impairment based on Z-score (n=24 993).**

|  | **Normal** | **Mild** | **Moderate** | **Moderate-severe** | **Severe** | **Very severe** |
| --- | --- | --- | --- | --- | --- | --- |
|  | **(n=21 894)** | **(n=1 054)** | **(n=925)** | **(n=523)** | **(n=471)** | **(n=126)** |
|  |  |  |  |  |  |  |
| Age (years), mean (SD) | 58.7 (7.3) | 59.2 (7.1) | 59.7 (6.9) | 59.9 (7.3) | 60.2 (7.2) | 57.6 (6.6) |
| Age (years), % |  |  |  |  |  |  |
| *< 50* | 15.9 | 12.9 | 10.6 | 12.0 | 10.8 | 17.5 |
| *50-59* | 38.6 | 38.7 | 38.3 | 35.4 | 35.9 | 42.9 |
| *60-69* | 40.7 | 44.5 | 46.0 | 48.4 | 48.4 | 39.7 |
| *≥ 70* | 4.8 | 3.9 | 5.1 | 4.9 | 4.9 | 0.0 |
| Women, % | 54.3 | 50.3 | 48.9 | 48.0 | 43.5 | 46.0 |
| Country, % |  |  |  |  |  |  |
| *Czech Republic* | 26.2 | 35.2 | 33.2 | 29.4 | 31.8 | 30.9 |
| *Russia* | 27.2 | 23.0 | 25.9 | 32.5 | 34.2 | 44.5 |
| *Poland* | 17.9 | 21.0 | 22.4 | 20.5 | 18.1 | 13.5 |
| *Lithuania* | 29.7 | 20.8 | 18.5 | 17.6 | 15.9 | 11.1 |
| Occupational status, % |  |  |  |  |  |  |
| *Employed* | 40.3 | 33.8 | 31.9 | 27.5 | 23.2 | 31.7 |
| *Retired/employed* | 13.6 | 10.9 | 9.8 | 10.9 | 9.6 | 4.0 |
| *Retired/unemployed* | 41.1 | 50.0 | 52.8 | 54.9 | 60.3 | 54.8 |
| *Unemployed* | 5.0 | 5.3 | 5.5 | 6.7 | 6.8 | 9.5 |
| Smoking status, % |  |  |  |  |  |  |
| *Never* | 54.7 | 39.2 | 38.2 | 32.2 | 32.1 | 32.5 |
| *Past smoker* | 21.5 | 23.1 | 23.7 | 23.4 | 24.0 | 19.8 |
| *Current, < 1 cigarette* | 1.9 | 1.5 | 1.7 | 3.3 | 2.1 | 1.6 |
| *Current, ≥ 1 cigarette* | 21.8 | 36.2 | 36.4 | 41.2 | 41.8 | 46.1 |
| Smoking category^a^, % |  |  |  |  |  |  |
| *Light* | 47.9 | 39.4 | 36.1 | 39.4 | 36.3 | 27.7 |
| *Moderate* | 42.8 | 48.4 | 50.5 | 49.2 | 50.8 | 66.3 |
| *Heavy* | 9.3 | 12.2 | 13.4 | 11.4 | 12.9 | 6.0 |
| Alcohol consumption^b^, % |  |  |  |  |  |  |
| *Never* | 26.4 | 29.0 | 26.3 | 29.7 | 26.7 | 29.4 |
| *<1/monthly* | 28.8 | 24.9 | 29.0 | 26.4 | 27.6 | 27.0 |
| *1-3/monthly* | 21.0 | 21.9 | 17.1 | 18.7 | 17.5 | 18.3 |
| *1-4/weekly* | 19.0 | 17.6 | 20.6 | 19.6 | 22.2 | 19.8 |
| *≥5/weekly* | 4.8 | 6.6 | 7.0 | 5.6 | 6.0 | 5.5 |
| Deprivation range^c^, mean (SD) | 2.1 (2.9) | 2.4 (3.1) | 2.6 (3.2) | 3.0 (3.5) | 2.9 (3.2) | 3.5 (3.4) |
| Physical activity moderate^d^, mean (SD) | 15.4 (11.4) | 14.2 (11.2) | 14.1 (11.9) | 14.0 (11.3) | 13.9 (12.0) | 15.0 (12.3) |
| Physical activity vigorous^e^, mean (SD) | 3.7 (5.6) | 3.8 (6.4) | 3.1 (4.9) | 3.3 (5.6) | 3.7 (6.9) | 2.5 (4.9) |
| BMI, mean (SD), kg/m^2^ | 28.5 (4.9) | 29.4 (5.6) | 29.4 (6.0) | 29.4 (6.3) | 28.5 (5.9) | 27.5 (6.6) |
| **Comorbidities, %** |  |  |  |  |  |  |
| ***Cardiovascular diseases*** |  |  |  |  |  |  |
| *Hypertension* | 63.8 | 71.2 | 70.7 | 73.9 | 74.6 | 66.7 |
| *Myocardial infarction* | 6.5 | 9.8 | 12.2 | 10.1 | 11.4 | 10.4 |
| *Ischemic heart disease* | 12.1 | 15.1 | 18.2 | 19.2 | 15.9 | 20.0 |
| *Stroke* | 3.5 | 6.3 | 5.4 | 5.5 | 4.5 | 6.5 |
| ***Lung diseases*** |  |  |  |  |  |  |
| *COPD* | 14.6 | 22.2 | 31.4 | 37.1 | 47.0 | 42.4 |
| *Asthma* | 3.2 | 7.2 | 11.4 | 16.9 | 23.1 | 17.6 |
| *Cough (>3 months)* | 13.7 | 20.2 | 25.7 | 37.3 | 45.5 | 39.7 |
| *Chest pain (>3 months)* | 13.0 | 20.3 | 23.1 | 31.4 | 39.4 | 32.0 |
| ***Any type of cancer*** | 5.3 | 4.6 | 6.2 | 7.2 | 5.8 | 4.0 |
| ***Other diseases*** |  |  |  |  |  |  |
| *Diabetes* | 8.2 | 13.8 | 14.1 | 11.9 | 13.6 | 4.8 |
| *Any type of surgery* | 1.2 | 0.8 | 1.3 | 0.8 | 1.5 | 2.4 |
| ***Spirometry, mean (SD)*** |  |  |  |  |  |  |
| *FEV1* | 2.7 (0.7) | 2.0 (0.5) | 1.8 (0.5) | 1.6 (0.4) | 1.2 (0.4) | 0.8 (0.3) |
| *FVC* | 3.3 (0.8) | 2.7 (0.7) | 2.5 (0.8) | 2.4 (0.8) | 2.2 (0.8) | 2.1 (1.0) |
| *Z-score^f^* | -0.1 (0.9) | -1.8 (0.1) | -2.2 (0.1) | -2.7 (0.1) | -3.4 (0.3) | -4.4 (0.4) |

BMI, body mass index; COPD, chronic obstructive pulmonary disease; FEV1, forced expiratory volume in 1 second; FVC, forced vital capacity.

^a^ Smoking category ((current or past heavy smoker (>30 cigarettes per day), moderate smoker (11 - 29 cigarettes per day), or light smoker (<10 cigarettes per day)).

^b^ Alcohol consumption (never, graduated frequency from 1-3 drinks monthly or 1-5 drinks weekly).

^c^Deprivation scale (graded from 1 as a least deprived up to 12 as a most deprived).

^d^Number of hours per week undertaken by household domain physical activity (e.g., housework, gardening, maintenance of the house etc).

^e^Number of hours of vigorous physical activity per week (e.g., sports, play games and hiking)

^f^The reference values from the Global Lung Initiative (GLI) with threshold point below lower limit of normal (-1.645)

**Table S3.** **Survival data by country.**

| **Country** | **No. of persons** | **No. of deaths** | **Person-years of follow-up** | **Deaths per 100 person-years** | **Mean Follow-up time (years)** | **Median Follow-up time (years)** |
| --- | --- | --- | --- | --- | --- | --- |
| *Czech Republic* | 6 768 | 1 612 | 106 043 | 1.52 (1.45-1.60) | 15.7 | 16.9 |
| *Russia* | 6 822 | 1 562 | 83 492 | 1.87 (1.78-1.97) | 12.3 | 13.5 |
| *Poland* | 4 559 | 793 | 57 302 | 1.38 (1.29-1.48) | 12.5 | 13.5 |
| *Lithuania* | 6 844 | 1 210 | 75 252 | 1.61 (1.52-1.70) | 10.9 | 11.6 |

**Table S4. Association between degree of lung function impairment and all-cause mortality by type of FVC predicted.**

| **Type of groups** | **No. of persons** | **No. of deaths** | **Person-years of follow-up** | | **Deaths per 100 person-years (95% CI)** | | **Model 1 adjusted HR† (95% CI)** | | | **Model 2 Adjusted HR‡ (95% CI)** |
| --- | --- | --- | --- | --- | --- | --- | --- | --- | --- | --- |
| ***FVC % predicted impairment groups (NHANES)**** | | | |  | |  | |  |  |  |
| *Normal* | 18 962 | 3 317 | 248 111 | | 1.34 (1.29-1.38) | | 1.00 | | | 1.00 |
| *Mild* | 3 879 | 1 033 | 49 227 | | 2.10 (1.97-2.23) | | 1.51 (1.41-1.62) | | | 1.27 (1.18-1.37) |
| *Moderate* | 1 240 | 439 | 14 951 | | 2.94 (2.67-3.22) | | 2.00 (1.81-2.21) | | | 1.50 (1.35-1.67) |
| *Moderate-severe* | 386 | 175 | 4 336 | | 4.00 (3.48-4.68) | | 2.54 (2.18-2.96) | | | 1.84 (1.57-2.17) |
| *Severe* | 137 | 79 | 1 320 | | 5.99 (4.80-7.46) | | 3.99 (3.19-4.99) | | | 3.03 (2.41-3.82) |
| *Very severe* | 25 | 18 | 218 | | 8.25 (5.20-13.1) | | 4.49 (2.82-7.14) | | | 3.17 (1.98-5.06) |
| ***FVC impairment groups (Z-score)^§^*** | | | |  | |  | |  |  |  |
| *Normal* | 19 501 | 3 579 | 254 286 | | 1.41 (1.36-1.45) | | 1.00 | | | 1.00 |
| *Mild* | 2 024 | 510 | 25 869 | | 1.97 (1.81-2.15) | | 1.47 (1.34-1.62) | | | 1.24 (1.12-1.37) |
| *Moderate* | 1 796 | 485 | 22 711 | | 2.14 (1.95-2.33) | | 1.57 (1.43-1.73) | | | 1.28 (1.15-1.41) |
| *Moderate-severe* | 847 | 277 | 10 248 | | 2.70 (2.40-3.04) | | 2.03 (1.79-2.29) | | | 1.54 (1.35-1.75) |
| *Severe* | 633 | 248 | 7 298 | | 3.40 (3.00-3.85) | | 2.45 (2.15-2.79) | | | 1.77 (1.54-2.03) |
| *Very severe* | 158 | 78 | 1 677 | | 4.65 (3.72-5.81) | | 3.74 (2.99-4.68) | | | 2.93 (2.31-3.70) |

FEV1, forced expiratory volume in 1 second; CI, confidence interval; HR, hazard ratio.

*Sex-specific predicted values of FEV1 (FEV1%) standardized for age and height based on NHANES III equations.

*^§^*The reference values from the GLI 2012 z-score with threshold point below lower limit of normal (-1.645)

†Adjusted for age, sex and country.

‡Adjusted for age, sex, occupation and education; alcohol consumption, smoking status, level of physical activity and body mass index; history of hypertension, ischemic heart disease, myocardial infarction, stroke.

**Table S5. Association between degree of lung function impairment (FEV1% predicted) and all-cause mortality by country (follow-up restricted to 10 years).**

| **Type of groups** | **No. of persons** | **No. of deaths** | **Person-years of follow-up** | **Deaths per 100 person-years (95% CI)** | **Model 1 adjusted HR† (95% CI)** | **Model 2 adjusted HR‡ (95% CI)** |
| --- | --- | --- | --- | --- | --- | --- |
| ***Czech Republic*** |  |  |  |  |  |  |
| ***FEV1 % predicted impairment groups**** |  |  |  |  |  |  |
| *Normal* | 5 283 | 440 | 51 055 | 0.86 (0.78-0.95) | 1.00 | 1.00 |
| *Mild* | 831 | 113 | 7 875 | 1.43 (1.19-1.73) | 1.53 (1.24-1.88) | 1.21 (0.96-1.53) |
| *Moderate* | 366 | 65 | 3 380 | 1.92 (1.51-2.45) | 1.86 (1.43-2.41) | 1.39 (1.04-1.88) |
| *Moderate-severe* | 135 | 30 | 1 228 | 2.44 (1.71-3.49) | 2.41 (1.67-3.50) | 1.61 (1.05-2.48) |
| *Severe* | 110 | 31 | 958 | 3.24 (2.28-4.60) | 3.15 (2.19-4.52) | 2.25 (1.50-3.37) |
| *Very severe* | 29 | 9 | 244 | 3.68 (1.92-7.08) | 3.38 (1.70-6.71) | 2.59 (1.23-5.43) |
| ***Russia*** |  |  |  |  |  |  |
| ***FEV1 % predicted impairment groups**** |  |  |  |  |  |  |
| *Normal* | 5 621 | 744 | 52 589 | 1.41 (1.32-1.52) | 1.00 | 1.00 |
| *Mild* | 595 | 127 | 5 346 | 2.38 (2.00-2.83) | 1.42 (1.18-1.72) | 1.12 (0.93-1.36) |
| *Moderate* | 262 | 83 | 2 163 | 3.84 (3.09-4.76) | 2.38 (1.89-2.99) | 1.84 (1.46-2.32) |
| *Moderate-severe* | 163 | 59 | 1 319 | 4.47 (3.47-5.77) | 2.56 (1.94-3.38) | 1.82 (1.38-2.39) |
| *Severe* | 116 | 40 | 962 | 4.16 (3.05-5.67) | 2.06 (1.50-2.82) | 1.51 (1.09-2.10) |
| *Very severe* | 46 | 25 | 316 | 7.92 (5.35-11.72) | 3.87 (2.47-6.05) | 2.82 (1.87-4.25) |
| ***Poland*** |  |  |  |  |  |  |
| ***FEV1 % predicted impairment groups**** |  |  |  |  |  |  |
| *Normal* | 3 650 | 324 | 35 054 | 0.92 (0.82-1.03) | 1.00 | 1.00 |
| *Mild* | 504 | 81 | 4 719 | 1.72 (1.38-2.13) | 1.53 (1.25-1.87) | 1.20 (0.96-1.50) |
| *Moderate* | 234 | 45 | 2 143 | 2.10 (1.57-2.81) | 2.16 (1.57-2.97) | 1.75 (1.26-2.43) |
| *Moderate-severe* | 97 | 32 | 821 | 3.90 (2.76-5.51) | 3.56 (2.48-5.13) | 2.41 (1.63-3.57) |
| *Severe* | 59 | 20 | 478 | 4.18 (2.70-6.48) | 3.21 (2.01-5.13) | 2.23 (1.34-3.70) |
| *Very severe* | 14 | 8 | 102 | 7.86 (3.93-15.72) | 8.44 (4.27-16.68) | 4.81 (2.27-10.18) |
| ***Lithuania*** |  |  |  |  |  |  |
| ***FEV1 % predicted impairment groups**** |  |  |  |  |  |  |
| *Normal* | 5 952 | 702 | 56 462 | 1.24 (1.15-1.34) | 1.00 | 1.00 |
| *Mild* | 522 | 116 | 4 693 | 2.47 (2.06-2.97) | 1.96 (1.60-2.39) | 1.56 (1.27-1.92) |
| *Moderate* | 206 | 72 | 1 693 | 4.25 (3.38-5.36) | 3.08 (2.41-3.95) | 2.22 (1.73-2.86) |
| *Moderate-severe* | 92 | 37 | 728 | 5.09 (3.68-7.02) | 3.04 (2.12-4.36) | 2.36 (1.68-3.30) |
| *Severe* | 57 | 25 | 447 | 5.60 (3.78-8.28) | 3.23 (2.14-4.89) | 2.12 (1.40-3.20) |
| *Very severe* | 15 | 10 | 85 | 11.82 (6.36-21.97) | 6.40 (2.91-14.06) | 4.32 (2.27-8.23) |

FEV1, forced expiratory volume in 1 second; CI, confidence interval; HR, hazard ratio.

*Sex-specific predicted values of FEV1 (FEV1%) standardized for age and height based on NHANES III equations

†Adjusted for age and sex.

‡Adjusted for age, sex, occupation and education; alcohol consumption, smoking status, level of physical activity and body mass index; history of hypertension, ischemic heart disease, myocardial infarction, stroke.

**Figure S1.** **Kaplan-Meier survival curves by groups of lung function impairment.**


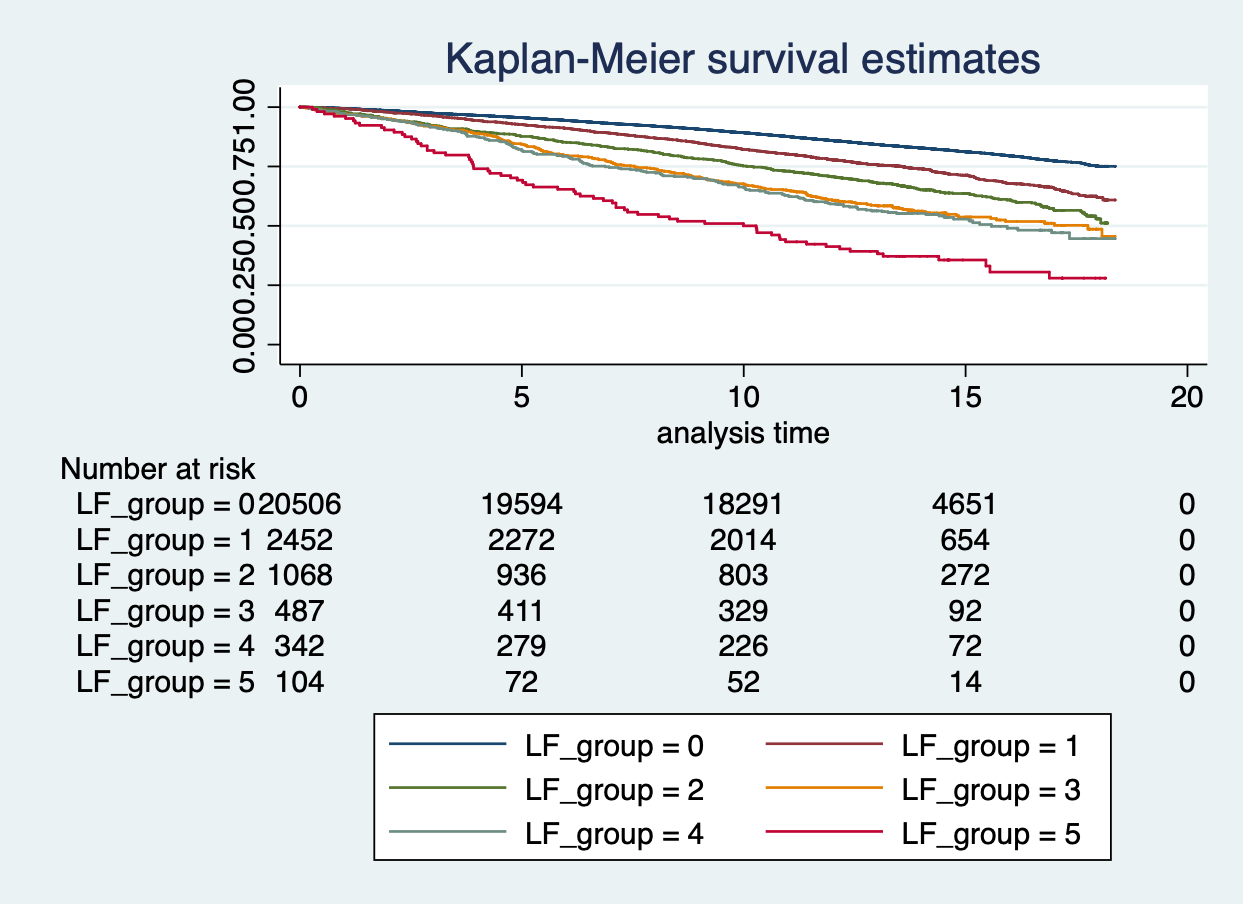

Supplement: Supplementary file 1 — Additional file 1: Table S1. Characteristics of the study sample by degree of lung function impairment based on (NHANES) III equations (n=24 993). Table S2. Characteristics of the study sample by degree of lung function impairment based on Z-score (n=24 993). Table S3. Survival data by country. Table S4. Association between degree of lung function impairment and all-cause mortality by type of FVC predicted. Table S5. Association between degree of lung function impairment (FEV1% predicted) and all-cause mortality by country (follow-up restricted to 10 years). Figure S1. Kaplan–Meier survival curves by groups of lung function impairment. [file 12931_2022_2057_MOESM1_ESM.docx]
